# Post‐GWAS Polygenic Risk Score: Utility and Challenges

**DOI:** 10.1002/jbm4.10411

**Published:** 2020-09-19

**Authors:** Tuan V Nguyen, John A Eisman

**Affiliations:** ^1^ Healthy Ageing Theme Garvan Institute of Medical Research Sydney Australia; ^2^ St Vincent's Clinical School UNSW Medicine, UNSW Sydney Australia; ^3^ School of Medicine Sydney University of Notre Dame Sydney Australia; ^4^ School of Biomedical Engineering University of Technology Sydney Australia

**Keywords:** DISEASES AND DISORDERS OF/RELATED TO BONE, OSTEOPOROSIS, GENETIC RESEARCH, HUMAN ASSOCIATION STUDIES, PRACTICE/POLICY‐RELATED ISSUES, FRACTURE RISK ASSESSMENT

## Abstract

Over the past decade, through genome‐wide association studies, more than 300 genetic variants have been identified to be associated with either BMD or fracture risk. These genetic variants are common in the general population, but they exert small to modest effects on BMD, suggesting that the utility of any single variant is limited. However, a combination of effect sizes from multiple variants in the form of the polygenic risk score (PRS) can provide a useful indicator of fracture risk beyond that obtained by conventional clinical risk factors. In this perspective, we review the progress of genetics of osteoporosis and approaches for creating PRSs, their uses, and caveats. Recent studies support the idea that the PRS, when integrated into existing fracture prediction models, can help clinicians and patients alike to better assess the fracture risk for an individual, and raise the possibility of precision risk assessment. © 2020 The Authors. *JBMR Plus* published by Wiley Periodicals LLC on behalf of American Society for Bone and Mineral Research.

Consider the following two 70‐year‐old women. Case 1 has femoral neck BMD of 0.72 g/cm^2^ (eg, nonosteoporosis), has no prior fracture, but fell once over the past 12 months. Case 2 has femoral neck BMD of 0.65 g/cm^2^ (eg, osteoporosis), has no prior fracture, and had no fall during the past 12 months. If the two women have genotypes that are associated with an osteoporosis phenotype, how can the genetic data help inform their fracture risk? Below, we review the genetic influence on osteoporosis phenotypes and the generation, as well as the application of the polygenic risk score (PRS) in individualized fracture risk assessment.

## The Genetics of Osteoporosis

The genetics of osteoporosis has, over the past five decades, evolved through five paradigms: heritability study, candidate gene study, genome‐wide association study (GWAS), and recently, the PRS and whole‐genome sequencing. Twin studies in the 1980s and 1990s showed that up to 80% of the variance in BMD was attributable to genetic factors.^(^ ^1^, ^2^ ^)^ Twin studies also showed that between 25% to 35% of the variance in the liability to fracture is heritable,^(^ ^3^, ^4^ ^)^ consistent with the observation that women with a familial history of hip fracture have a twofold increase in the risk of hip fracture.^(^ ^5^ ^)^ Moreover, genetic factors account for a large proportion of variance in recognized risk factors for fracture such as bone loss,^(^ ^6^ ^)^ quantitative ultrasound,^(^ ^7^ ^)^ and bone turnover markers.^(^ ^8^ ^)^ These lines of evidence have established that heredity is an important risk factor for osteoporosis and fracture risk.

Although the demonstration of hereditary effect on osteoporosis risk is relatively easy, the identification of specific genes that contribute to the risk has proven to be a formidable task. After a series of candidate gene studies, a number of “osteoporosis genes” (including, but not limited to, vitamin D receptor [VDR], collagen type 1 alpha 1 genes) have been identified.^(^ ^9^ ^)^ However, these candidate gene studies were marred by conflicting findings, limited reproducibility, and possibly false‐positives,^(^ ^10^ ^)^ which were perhaps based mainly on the lack of statistical power.^(^ ^11^ ^)^

Instead of focusing on a biologically plausible candidate gene, a GWAS offers a hypothesis‐free method of searching for putative genes in the entire genome without any assumptions about the location and functional significance of loci or their products.^(^ ^12^ ^)^ The thinking behind a GWAS is actually the “common disease–common variant” hypothesis, which postulates that the genetic component of common diseases (such as osteoporosis) is made up of a large number of putative alleles that are common (>5%) in the general population.^(^ ^13^ ^)^

Although it is a hypothesis‐free approach, a GWAS has been successful in identifying multiple variants that are associated with BMD or fracture risk. A significant breakthrough was reported in a seminal study that analyzed 301,019 variants in ~14,000 individuals of White background, and discovered 77 variants that were associated with BMD at the genome‐wide significance level.^(^ ^14^ ^)^ Some of these variants are located close to or within genes that are known to have important biologic roles in bone metabolism, such as RANK, RANKL, osteoprotegerin (OPG), estrogen receptor 1 (ESR1), and zinc finger and BTB domain containing 40 (ZBTB40). Apart from those variants, the study also identified several variants in and around the VDR and low‐density lipoprotein receptor‐related protein 5 (LRP5) genes that had been intensively examined in candidate gene studies.

Another seminal study comprised of 81,949 cases and 102,444 controls, largely of White background, identified 56 loci that were associated with BMD and 13 SNPs associated with fracture.^(^ ^15^ ^)^ Several of these loci or SNPs also cluster within or near the RANK–RANKL–OPG system, mesenchymal stem cell differentiation, endochondral ossification, and Wnt signaling pathways. A more recent study using the resource of UK Biobank identified 518 loci associated with heel ultrasound measurements, of which 301 were new loci.^(^ ^16^ ^)^ The list of genetic variants identified so far is unlikely the final list, as ongoing studies are likely to identify more variants that contribute to the susceptibility to osteoporosis and fracture.

The identified variants have two common characteristics: They are carried by a large proportion of people, and they confer modest effect sizes. Indeed, the frequency of the minor allele for all variants discovered so far ranged mostly between 5% and 45% in the general population. Moreover, the strength of association between these variants and traits is modest or very modest. Most of the variants that are associated with fracture risk had modest odds ratios (ORs; ie, <1.2). For example, the 77 variants in the first major GWAS accounted for only 3% of the total variance in hip and spinal BMD,^(^ ^14^ ^)^ and the 518 variants in the UK Biobank study accounted for 20% of the variance in heel ultrasound measurement. In terms of fracture association, virtually all variants confer modestly increased odds of fracture by a few percentage points, mostly closer to 5% and albeit some rare loci up to 20%.

The modest effect size of genetic variants implies that the predictive value of any single genetic variant is likely limited; this is entirely expected by simple epidemiologic principle.^(^ ^17^ ^)^ For instance, for a genetic variant with OR between 1.1 and 1.2, and assuming that the 5‐year incidence of fracture is 10%, the area under the ROC curve (AUC) associated with this genetic variant is expected to be 0.52 to 0.55. Thus, any single variant of and by itself is unlikely to be useful for prediction.

On the other hand, a combination of multiple genetic variants, even with modest effect sizes, could be useful. Theoretically, it can be shown that for a given number of variants, the discriminatory power (eg, AUC) increases proportionally to the effect sizes.^(^ ^18^ ^)^ For example, for a combination of 50 genetic variants, each with an OR = 1.1, the AUC value is expected to be 0.63; however, if each variant has an OR = 1.2, the AUC is expected to be 0.73. From this simulation, it can also be inferred that a model with 500 variants, each with OR = 1.1, can yield an AUC >0.80 (Table 1). The idea of combining multiple variants gives rise to a construct known as the polygenic risk score, which is poised to improve patient outcomes via precision medicine,^(^ ^19^ ^)^ but raises concerns of health disparity between ethnicities.^(^ ^20^ ^)^

**Table 1:** Utility of Genetic Profiling in Terms of AUC and Percentage of Net Reclassification Improvement

Number of variants	AUC				
	OR = 1.10	OR = 1.15	OR = 1.20	OR = 1.25	OR = 1.30
10	0.557	0.586	0.611	0.635	0.659
50	0.630	0.682	0.733	0.769	0.805
100	0.682	0.743	0.794	0.841	0.870
500	0.830	0.898	0.936	0.953	0.967
1000	0.895	0.943	0.966	0.977	0.983

Notes. Results were obtained by simulation with the following parameters: gene frequency = 0.5, risk threshold = 0.2, and 5‐year incidence of fracture = 0.1. AUC = Area under the receiver operating characteristic curve; OR = odds ratio.

## The Polygenic Risk Score

The PRS can be defined as a quantitative index of the genetic burden related to a specific disorder and is specific to an individual. Operationally, there are several ways to create a PRS. The simplest approach is to assign a risk value of 0 if an individual is a noncarrier of a risk allele, 0.5 or 1 if a carrier, and 1 or 2 if homozygous for that allele, and then sum the score across variants for the individual. This simple approach implicitly assumes that all variants contribute equally to the trait variation, which is unlikely true in most real‐world situations. Therefore, a better approach is to sum the trait‐associated alleles weighted by their effect sizes. The trait‐associated or risk allele is defined as an allele that is more common in cases than controls. The effect size can be a regression coefficient (for quantitative trait) or log OR (for categorical traits). In either approach, because the PRS is aggregated from multiple variants and effect sizes, it is likely to be unique to an individual. Moreover, given its aggregation nature, the PRS can be seen as an index of an individual's genetic liability to develop a disorder.

Statistically, the PRS tends to show a normal or approximately normal distribution in the general population. The difference in the PRS between those with and without a disorder is mainly governed by the mean, not the variance of the distribution.

The number of variants can range between a few and millions that are identified across loci from GWASs. Intuitively, only variants that are statistically robust at genome‐wide significance level should be included in the derivation of the PRS because variants that do not reach the significance level are unlikely to contribute to the variation in the trait of interest. However, in reality, some PRSs were constructed from many variants (eg, hundreds of thousands of variants) across the genome, even though they show very weak association with a trait. This practice of including a large number of variants is based on the view that many genuine associations are potentially missed because of inadequate power in the original GWAS.

Another approach to construct the PRS is to use knowledge of biological pathways or processes to select relevant variants. For example, one can select all variants that are involved with protein–protein interactions or involvement in signaling pathways. However, it remains to be shown whether pathway‐based PRSs can predict phenotypic variation more robustly than the “traditional” PRS, which is based on the totality of disease risk.

## What Can the Polygenic Risk Score Be Used For?

An obvious application of a PRS in clinical osteoporosis care is fracture risk assessment. At present, the assessment of fracture risk for an individual is primarily done using tools such as the Garvan fracture risk calculator,^(^ ^21^ ^)^ the fracture risk assessment tool (FRAX),^(^ ^22^ ^)^ and QFract software.^(^ ^23^ ^)^ Although these tools have proven useful for identifying high‐risk individuals, their predictive performance is a matter for improvement. In external validation studies, the AUC of the Garvan and FRAX models ranged between 0.61 and 0.85, with average being ~0.70,^(^ ^24^, ^25^, ^26^, ^27^ ^)^ a modest discrimination. Ideally, to be useful, predictive capacity would need to be high predictive (AUC ~0.80). Furthermore, FRAX tends to underestimate the risk of fracture by as much as 50%,^(^ ^24^ ^)^ in part because factors that are associated with higher risk are also associated with earlier mortality for which the estimated risk was discounted. Thus, there is room for further improvement in fracture prediction, and genetic factors have emerged as an important for fracture risk prediction.

Although none of the existing fracture prediction tools incorporates genetic data, recent studies have suggested that the PRS can be a useful addition. We have created a PRS called osteogenomic profile,^(^ ^28^ ^)^ which is based on 62 variants that are associated with BMD.^(^ ^14^ ^)^ We found that each unit increase in the PRS was associated with a hazard ratio of 1.20 (95% CI, 1.04–1.38) for fracture, independent of age, prior fracture, and falls.^(^ ^28^ ^)^ Importantly, when the PRS was included in the existing Garvan fracture risk calculator model,^(^ ^29^ ^)^ the reclassification of fracture versus nonfracture was significantly improved.^(^ ^28^ ^)^ In the MrOS (Osteoporotic Fractures in Men study) cohort, a PRS constructed from 63 genetic BMD‐associated variants was also associated with the risk of total fracture.^(^ ^30^ ^)^ In postmenopausal women of Korean background, a PRS constructed from 39 variants improved the precision of nonertebral fracture prediction in the general population,^(^ ^31^ ^)^ as well as in patients on bisphosphonate.^(^ ^31^, ^32^ ^)^ QUS is associated with fracture,^(^ ^33^ ^)^ and the PRS generated from QUS can also help identify individuals at risk of fracture or osteoporosis.^(^ ^34^, ^35^ ^)^ Taken together, a PRS constructed from BMD‐associated variants could be used as a genetic factor for fracture prediction.

The PRS can also be used for assessing bone loss in an individual. Bone loss is highly variable between individuals, not simply because of measurement error, but also because of genetic factors.^(^ ^6^ ^)^ Although specific genes for bone loss have not been identified, a PRS has been shown to be associated with bone loss in postmenopausal women.^(^ ^36^ ^)^ In a longitudinal study in which BMD of 860 postmenopausal women had been monitored for up to 20 years, each unit higher PRS was associated with a 0.21% (standard error, 0.10) higher annual rate of bone loss at the femoral neck. Of note, this association was independent of baseline BMD and age. Moreover, each unit increase of PRS was associated with 41% odds (95% CI, 1.07–1.87) of rapid bone loss (defined as loss >1.2%/year). Thus, the PRS could be used as an additional means for predicting bone loss in an individual.^(^ ^36^ ^)^

The PRS can be considered an index of family history. It is well‐known that a family history of fracture, especially a family history of hip fracture, is a risk factor for fracture: Daughters of mothers with a history of hip fracture have lower BMD than those whose mothers did not have a hip fracture.^(^ ^37^ ^)^ Women with a familial history of hip fracture have a twofold increase in the risk of hip fracture.^(^ ^5^ ^)^ However, family history is an unreliable measure, partly because of recall bias and in part because it relies on older family members surviving to ages that mean they can contribute useful information for risk. Thus, despite its potential, family history poorly captures the polygenic nature of risk. By contrast, the PRS represents an individual's overall genomic burden that is heritable, making the PRS an attractive quantitative index of family history.

## What the Polygenic Risk Score Is Not

The PRS is not a diagnostic test. Like other risk assessment tools, the PRS can only indicate the risk of fracture, but cannot categorically establish whether an individual will or will not have a fracture. However, the advantage of risk assessment via the PRS is that it allows a life‐time prediction well before the onset of fracture. Although there is no “genetic therapy” for individuals at high risk of fracture, this salient lifetime prediction may raise immediacy of primary and secondary prevention behaviors in those at heightened genetic risk.

The PRS alone is not informative of fracture status. That is, it cannot reliably discriminate between those who will from those who will not have a fracture. The PRS must and should be used in conjunction with established risk factors (eg, BMD, fall, personal history of fracture) for assessing fracture risk. Still, the PRS could be useful for the stratification of individuals in the general population, which can be useful for public health intervention.

## Caveats

Any scientifically useful measure should satisfy two basic criteria: content validity and criterion validity. Content validity refers to the extent to which the measure is representative of the entire domain or content the measure seeks to reflect. Criterion validity is the correlation between the new measure and an established method (eg, the gold standard). Any use or interpretation of the PRS must be considered in the context of these two types of validity.

In the PRS context, genetic liability is the domain. The PRS, which is constructed from a selected set of genetic variants, could not capture the totality of genetic liability. As mentioned above, different PRSs have been constructed using different approaches and criteria. The selection of variants has largely been based on *P*‐value thresholds, so it is unlikely that they capture the totality of genetic liability. Logically, the more variants to be included in the PRS (usually by relaxing *P*‐value criteria) the better content validity it is, and this has been shown in simulation as well as empirical studies.^(^ ^38^ ^)^ Moreover, rare variants (ie, allelic frequencies <1%) that are not currently identified by GWASs may account for a significant proportion of variance in BMD,^(^ ^39^ ^)^ but they are not included in the derivation of the PRS. At present, most PRSs account for a small proportion of genetic variance of BMD or fracture risk, and their content validity is an open question.

The criterion validity of the PRS also requires more consideration. BMD has been the gold standard for assessing fracture risk, and BMD has been shown to be valid in terms of measured content and its biology criterion. In the PRS context, the correlation between a PRS and BMD is low, with most correlation coefficients being <0.20, suggesting that PRSs have a low level of criterion validity.

The PRS is, as expected from the operational definition, sensitive to allele frequency and effect size of variants. Allele frequency is known to be different between ethnicities, such that an allele may be common in one ethnicity, yet may be rare in another ethnicity. Furthermore, the effect size associated with an allele can be different between ethnicities because of interaction with environmental factors. Furthermore, population history (eg, effective population size, immigration, and inbreeding) and inheritance patterns may be different between ethnic groups. Thus, a PRS constructed from an ethnicity may not be applicable to another ethnicity. At present, most PRSs in the field of osteoporosis were derived from White populations, and these PRSs may not be applicable to non‐White populations.

It is now increasingly recognized that many chronic diseases are biologically linked such that they form a network.^(^ ^40^ ^)^ The diseases within a network may share common sets of genetic variants. For instance, osteoporosis and obesity are known to be linked, and eight genetic variants at the TBX15 gene were associated with both body mass and estimated BMD.^(^ ^41^ ^)^ It has been suggested that genes associated with morphological phenotypes, such as bone structure, evolve more slowly than those associated with physiological phenotypes, such as bone turnover markers.^(^ ^42^ ^)^ The implication of these complex relationships is that the regression weight associated with a variant may be different across diseases and environmental factors, but this possibility is not captured by a phenotype‐specific PRS.

Another caveat of the PRS is its assumption of additive effects. Because the PRS is the sum of weighted effect alleles, it implicitly assumes that the effects of all variants are additive. However, there is no logical reason to suggest that the effects are independent; it is highly likely that the effect of one variant is dependent on the other's effect (ie, epistatic effects or gene–gene interaction), and this would introduce bias into the risk estimates. However, current methodology and sample size do not allow a complete dissection of higher‐order gene–gene interactions between genes. Nevertheless, in the presence of gene–gene interactions, the simple additive PRS can be biased.

Another concern with the PRS is its cost effectiveness. Although there have been no studies of the economic cost of PRS in osteoporosis, studies in the field of cancer^(^ ^43^ ^)^ and cardiovascular disease^(^ ^44^ ^)^ suggest that the PRS is cost effective in the prevention or improved management of disease. At present, it is quite feasible to generate the PRS with hundreds of thousands or even millions of SNPs for less than $100. Thus, in the long run, the implementation of the PRS into clinical practice will be technically and economically feasible.

Back to the two cases, what can the PRS contribute to the prediction of their fracture risk. The Garvan fracture risk calculator predicts that the two women have the same 10‐year risk of fracture (24%), which may not be indicated for treatment. However, if both women have a PRS in the top 5% percentile, then their 10‐year risk of fracture is now 32%, which may be indicated for treatment. Thus, knowing their genetic information in the form of a PRS can potentially change the indication of treatment.

In summary, the PRS has emerged as a useful measure of genetic propensity to a trait at the individual level that has multiple applications. In the field of osteoporosis, the PRS could be used for individualized fracture risk assessment in conjunction with existing prediction tools, prediction of bone loss, and as a quantitative index of family history. The PRS could also be used for risk stratification in the general population. However, the application of the PRS has to be considered in the context of its potential limitations concerning content and criterion validity and underlying assumption of additive effects. In the era of post‐GWAS, when health care will be strongly influenced by genomics and big data, the PRS, as a proxy measure of genetic liability, will likely have a place in clinical decision and precision medicine.

### Peer Review

The peer review history for this article is available at https://publons.com/publon/10.1002/jbm4.10411.

## References

[1] Pocock NA , Eisman JA , Hopper JL , Yeates MG , Sambrook PN , Eberl S . Genetic determinants of bone mass in adults. A twin study. J Clin Invest. 1987;80(3):706–10. 3624485 10.1172/JCI113125 PMC442294

[2] Nguyen TV , Howard GM , Kelly PJ , Eisman JA . Bone mass, lean mass, and fat mass: same genes or same environments? Am J Epidemiol. 1998;147(1):3–16. 9440393 10.1093/oxfordjournals.aje.a009362

[3] Deng HW , Chen WM , Recker S , et al. Genetic determination of Colles' fracture and differential bone mass in women with and without Colles' fracture. J Bone Miner Res. 2000;15(7):1243–52. 10893672 10.1359/jbmr.2000.15.7.1243

[4] Michaelsson K , Melhus H , Ferm H , Ahlbom A , Pedersen NL . Genetic liability to fractures in the elderly. Arch Intern Med. 2005;165(16):1825–30. 16157825 10.1001/archinte.165.16.1825

[5] Cummings SR , Nevitt MC , Browner WS , et al. Risk factors for hip fracture in white women. Study of Osteoporotic Fractures Research Group. N Engl J Med. 1995;332(12):767–73. 7862179 10.1056/NEJM199503233321202

[6] Makovey J , Nguyen TV , Naganathan V , Wark JD , Sambrook PN . Genetic effects on bone loss in peri‐ and postmenopausal women: a longitudinal twin study. J Bone Miner Res. 2007;22(11):1773–80. 17620052 10.1359/jbmr.070708

[7] Howard GM , Nguyen TV , Harris M , Kelly PJ , Eisman JA . Genetic and environmental contributions to the association between quantitative ultrasound and bone mineral density measurements: a twin study. J Bone Miner Res. 1998;13(8):1318–27. 9718201 10.1359/jbmr.1998.13.8.1318

[8] Tokita A , Kelly PJ , Nguyen TV , et al. Genetic influences on type I collagen synthesis and degradation: further evidence for genetic regulation of bone turnover. J Clin Endocrinol Metab. 1994;78(6):1461–6. 8200950 10.1210/jcem.78.6.8200950

[9] Ralston SH , de Crombrugghe B . Genetic regulation of bone mass and susceptibility to osteoporosis. Genes Dev. 2006;20(18):2492–506. 16980579 10.1101/gad.1449506

[10] Nguyen TV . Pharmacogenetics of anti‐resorptive therapy efficacy: a Bayesian interpretation. Osteoporos Int. 2005;16(8):857–60. 15685396 10.1007/s00198-004-1807-y

[11] Huang QY , Recker RR , Deng HW . Searching for osteoporosis genes in the post‐genome era: progress and challenges. Osteoporos Int. 2003;14(9):701–15. 12904838 10.1007/s00198-003-1445-9

[12] Wang WY , Barratt BJ , Clayton DG , Todd JA . Genome‐wide association studies: theoretical and practical concerns. Nat Rev Genet. 2005;6(2):109–18. 15716907 10.1038/nrg1522

[13] Reich DE , Lander ES . On the allelic spectrum of human disease. Trends Genet. 2001;17(9):502–10. 11525833 10.1016/s0168-9525(01)02410-6

[14] Styrkarsdottir U , Halldorsson BV , Gretarsdottir S , et al. Multiple genetic loci for bone mineral density and fractures. N Engl J Med. 2008;358(22):2355–65. 18445777 10.1056/NEJMoa0801197

[15] Estrada K , Styrkarsdottir U , Evangelou E , et al. Genome‐wide meta‐analysis identifies 56 bone mineral density loci and reveals 14 loci associated with risk of fracture. Nat Genet. 2012;44(5):491–501. 22504420 10.1038/ng.2249 PMC3338864

[16] Morris JA , Kemp JP , Youlten SE , et al. An atlas of genetic influences on osteoporosis in humans and mice. Nat Genet. 2019;51(2):258–66. 30598549 10.1038/s41588-018-0302-x PMC6358485

[17] Holtzman NA , Marteau TM . Will genetics revolutionize medicine? N Engl J Med. 2000;343(2):141–4. 10891526 10.1056/NEJM200007133430213

[18] Pepe MS , Gu JW , Morris DE . The potential of genes and other markers to inform about risk. Cancer Epidemiol Biomarkers Prev. 2010;19(3):655–65. 20160267 10.1158/1055-9965.EPI-09-0510 PMC2836397

[19] Sugrue LP , Desikan RS . What are polygenic scores and why are they important? JAMA. 2019;321(18):1820–1. 30958510 10.1001/jama.2019.3893

[20] Martin AR , Kanai M , Kamatani Y , Okada Y , Neale BM , Daly MJ . Clinical use of current polygenic risk scores may exacerbate health disparities. Nat Genet. 2019;51(4):584–91. 30926966 10.1038/s41588-019-0379-x PMC6563838

[21] Nguyen ND , Frost SA , Center JR , Eisman JA , Nguyen TV . Development of a nomogram for individualizing hip fracture risk in men and women. Osteoporos Int. 2007;18(8):1109–17. 17370100 10.1007/s00198-007-0362-8

[22] Kanis JA , Johnell O , Oden A , Johansson H , McCloskey E . FRAX and the assessment of fracture probability in men and women from the UK. Osteoporos Int. 2008;19(4):385–97. 18292978 10.1007/s00198-007-0543-5 PMC2267485

[23] Hippisley‐Cox J , Coupland C . Derivation and validation of updated QFracture algorithm to predict risk of osteoporotic fracture in primary care in the United Kingdom: prospective open cohort study. BMJ. 2012;344:e3427. 22619194 10.1136/bmj.e3427

[24] Bolland MJ , Siu AT , Mason BH , et al. Evaluation of the FRAX and Garvan fracture risk calculators in older women. J Bone Miner Res. 2011;26(2):420–7. 20721930 10.1002/jbmr.215

[25] Pluskiewicz W , Adamczyk P , Franek E , et al. Ten‐year probability of osteoporotic fracture in 2012 Polish women assessed by FRAX and nomogram by Nguyen et al.‐conformity between methods and their clinical utility. Bone. 2010;46(6):1661–7. 20156606 10.1016/j.bone.2010.02.012

[26] Pluskiewicza W , Adamczykb P , Franekc E , et al. Conformity between 10‐year probability of any osteoporotic fracture assessed by FRAX and nomogram by Nguyen et al. Bone. 2009;44(Suppl 2):S229–S30. 10.1016/j.bone.2010.02.012 20156606

[27] Sandhu SK , Nguyen ND , Center JR , Pocock NA , Eisman JA , Nguyen TV . Prognosis of fracture: evaluation of predictive accuracy of the FRAX algorithm and Garvan nomogram. Osteoporos Int. 2010 May;21(5):863–71. 19633880 10.1007/s00198-009-1026-7

[28] Ho‐Le TP , Center JR , Eisman JA , Nguyen HT , Nguyen TV . Prediction of bone mineral density and fragility fracture by genetic profiling. J Bone Miner Res. 2017;32(2):285–93. 27649491 10.1002/jbmr.2998

[29] Nguyen ND , Frost SA , Center JR , Eisman JA , Nguyen TV . Development of prognostic nomograms for individualizing 5‐year and 10‐year fracture risks. Osteoporos Int. 2008;19(10):1431–44. 18324342 10.1007/s00198-008-0588-0

[30] Eriksson J , Evans DS , Nielson CM , et al. Limited clinical utility of a genetic risk score for the prediction of fracture risk in elderly subjects. J Bone Miner Res. 2015;30(1):184–94. 25043339 10.1002/jbmr.2314 PMC4281709

[31] Lee SH , Lee SW , Ahn SH , et al. Multiple gene polymorphisms can improve prediction of nonvertebral fracture in postmenopausal women. J Bone Miner Res. 2013;28(10):2156–64. 23572424 10.1002/jbmr.1955

[32] Lee SH , Cho EH , Ahn SH , et al. Prediction of future osteoporotic fracture occurrence by genetic profiling: a 6‐year follow‐up observational study. J Clin Endocrinol Metab. 2016 Mar;101(3):1215–24. 26756118 10.1210/jc.2015-3972

[33] Moayyeri A , Adams JE , Adler RA , et al. Quantitative ultrasound of the heel and fracture risk assessment: an updated meta‐analysis. Osteoporos Int. 2012;23(1):143–53. 22037972 10.1007/s00198-011-1817-5

[34] Kim SK . Identification of 613 new loci associated with heel bone mineral density and a polygenic risk score for bone mineral density, osteoporosis and fracture. PLoS One. 2018;13(7):e0200785. 30048462 10.1371/journal.pone.0200785 PMC6062019

[35] Manousaki D , Forgetta V , Keller‐Baruch J , et al. A polygenic risk score as a risk factor for medication‐associated fractures. J Bone Miner Res. 2020 Jun 8. 10.1002/jbmr.4104. Online ahead of print. 32511779

[36] Ho‐Le TP , Pham HM , Center JR , Eisman JA , Nguyen HT , Nguyen TV . Prediction of changes in bone mineral density in the elderly: contribution of "osteogenomic profile". Arch Osteoporos. 2018;13(1):68. 29931598 10.1007/s11657-018-0480-2

[37] Seeman E . Reduced bone density in women with fractures: contribution of low peak bone density and rapid bone loss. Osteoporos Int. 1994;4(Suppl 1):15–25. 8081052 10.1007/BF01623430

[38] Chatterjee N , Wheeler B , Sampson J , Hartge P , Chanock SJ , Park JH . Projecting the performance of risk prediction based on polygenic analyses of genome‐wide association studies. Nat Genet. 2013;45(4):400–5 5e1‐3. 23455638 10.1038/ng.2579 PMC3729116

[39] Gibson G . Rare and common variants: twenty arguments. Nat Rev Genet. 2012;13(2):135–45. 22251874 10.1038/nrg3118 PMC4408201

[40] Goh KI , Cusick ME , Valle D , Childs B , Vidal M , Barabasi AL . The human disease network. Proc Natl Acad Sci U S A. 2007;104(21):8685–90. 17502601 10.1073/pnas.0701361104 PMC1885563

[41] Zhou Y , Wu K , Shen H , Zhang J , Deng HW , Zhao LJ . Geographical differences in osteoporosis, obesity, and sarcopenia related traits in white American cohorts. Sci Rep. 2019;9(1):12311. 31444391 10.1038/s41598-019-48734-9 PMC6707235

[42] Park S , Yang JS , Kim J , et al. Evolutionary history of human disease genes reveals phenotypic connections and comorbidity among genetic diseases. Sci Rep. 2012;2:757. 23091697 10.1038/srep00757 PMC3477654

[43] Callender T , Emberton M , Morris S , et al. Polygenic risk‐tailored screening for prostate cancer: a benefit‐harm and cost‐effectiveness modelling study. PLoS Med. 2019;16(12):e1002998. 31860675 10.1371/journal.pmed.1002998 PMC6924639

[44] Hynninen Y , Linna M , Vilkkumaa E . Value of genetic testing in the prevention of coronary heart disease events. PLoS One. 2019;14(1):e0210010. 30645616 10.1371/journal.pone.0210010 PMC6333335

